# Effects of Physical Exercise on Women with Disabilities in South Korea: A Meta-Analysis

**DOI:** 10.3390/ijerph182312791

**Published:** 2021-12-04

**Authors:** Yucheon Kim, Songyi Lee

**Affiliations:** 1 Counselling and Coaching Department, Graduate School, Dongguk University, Seoul 04620, Korea; yckim002@naver.com; 2 Dharma College, Dongguk University, Seoul 04620, Korea

**Keywords:** women, disabilities, physical exercise, meta-analysis, physical effects, psychological effects

## Abstract

This study is the first to conduct a comprehensive investigation of the effects of physical exercise on South Korean women with disabilities using the data of previous studies. This study performed a meta-analysis of the effect sizes of exercise programs for women with disabilities using Comprehensive Meta-Analysis 3.0 and a total of 16 papers with 154 participants. The largest effect sizes were found for changes in muscle strength (d = 2.407) for treatment effects, horseback riding (d = 3.080) for exercise type, 45–50 min (d = 3.080) for duration of a single exercise session, three times (d = 0.963) for frequency of exercise per week, 15 weeks (d = 1.974) for period of exercise and 45 times (d = 1.974) for total number of sessions. The results of this meta-analysis showed that exercise programs suitable for the individual-level characteristics of disabled individuals can and should be developed and implemented.

## 1. Introduction

People living with disabilities (PWDs) are regarded as the largest minority in the world, and they need to be included in public health programs [1]. Moreover, women living in low- and middle-income countries (LMICs) like India or Indonesia face additional barriers, including low decision-making power, gender-based violence (GBV) and lack of economic independence [2,3,4].

South Korea has been maintaining very rapid economic growth, and it is one of the world’s high-income countries. However, it still lags behind in terms of the welfare of the disabled [5]. South Korean expenditure on PWDs’ welfare is lower than that of Japan, at 0.98% of the GDP, and it remains at the bottom among OECD countries. In South Korean society, the quality of life of PWDs is poorer than that of non-disabled persons in many aspects, and that of female PWDs is particularly low [6,7].

There are 2,633,000 PWDs in South Korea (5.1% of the total population), which includes 1,110,000 women (42.2%), and this number is growing [8]. In general, female PWDs experience more discrimination and disadvantages than male PWDs [7,9]. Additionally, they are more vulnerable to emotional, physical and sexual abuse than women without disabilities [10,11] due to the intersectionality of gender and disability status. Further, society lacks consideration for the special needs of female PWDs [12].

Regarding physical exercise, female PWDs may face disadvantages due to the physiological challenges associated with maintaining their good looks and limited leisure activity opportunities [13,14]. For a long time, exercise was considered unsuitable for women, and their participation in it was limited; however, growing opportunities for women’s participation have increased interest in physical exercise in South Korean female PWDs [13,14]. Interest in such changes can be seen as a flow based on the ‘social model of disability’, which drew the existing phenomenon of equating disabilities with the concept of personal damage into the realm of socio-scientific analysis [15]. The theory of PWDs can be largely divided into a medical model and a social model. The medical model regards disabilities as products of personal tragedies or biological effects directly resulting from health conditions, which require medical protection provided in the form of individual treatments by experts [16]. In the social model, the cause of the problems faced by PWDs is not regarded to be a personal limitation, but rather a failure of society to provide adequate services and to ensure that the needs of PWDs are sufficiently considered within social organizations [17]. The fact that the interest in exercise of female PWDs began to appear in South Korea means that the lives of female PWDs are examined in the framework of the social model.

Although physical exercise, per se, is meaningful, the effects of exercise may vary according to the exercise type. Physical exercises include weight-bearing exercises, resistance exercises and aerobic exercises [18]. In weight-bearing exercises, the muscles and bones support one’s weight, and stimuli are applied against gravity. These stimuli are the same as those imposed on the feet, arms and legs in sports such as walking, mountain climbing, tennis, volleyball, dancing or aerobics. Aerobic exercises include swimming and cycling. Resistance exercises use free weights, equipment, props and one’s own weight. Weightlifting, push-ups and squats contract muscles and pull tendons to exert resistance on the bones, thereby affecting bone formation and reconstruction. Some of these exercise types are more appropriate for women’s physiological and psychological characteristics, and some also improve social functions.

In general, physical exercise has marked effects on the health and vitality of PWDs. It reduces health problems and has a positive effect on healthy functioning [19]. Physical exercise can provide opportunities for PWDs to socialize with people without disabilities and being able to compete equally with them instils pride, thereby improving social skills [20]. Physical exercise also affects physical self-efficacy and life satisfaction among people with physical disabilities [21,22]. Therefore, it can affect the physical and psychological aspects of health. This suggests that the effects of exercise should be considered for psychological as well as physical needs. However, PWDs tend to experience a sense of alienation or isolation during exercise, so a greater understanding of this issue may help promote exercise among this demographic [23].

However, other studies have reported different results regarding the effects of physical exercise among PWDs. Park [24] found that 6-month-long dance therapy had no significant effect on the emotional state of elderly (43–68 years old) participants with physical disabilities. Park and Park [25] showed that, among four obese adults with intellectual disabilities who participated in a yoga program, only one reported weight loss. These findings suggest that effects vary depending on the type of disability, age, sex and amount or type of physical exercise. PWDs may or may not be able to participate depending on the location, equipment and supports provided to help their movement [26,27]. Therefore, alleviating environmental constraints may enhance the effects of physical exercise.

Female PWDs have become increasingly interested in exercise in recent years; however, limited comprehensive analyses on the effects of physical exercise among this demographic have been conducted. There are also very few studies involving adequate surveys, analyses of exercise needs and assessments of participation in sports among female PWDs as an underprivileged population with special needs [28]. Therefore, the findings of prior studies need to be subjected to meta-analysis, a research synthesis technique that statistically summarizes prior research when studies are inconclusive due to inconsistent findings [29]. Accordingly, the present study analyzed the effect sizes of different studies based on the mean differences in exercise programs for female PWDs via meta-analysis utilizing Comprehensive Meta-Analysis (CMA) 3.0 software. Based on the findings, this study examines the way in which the effects of physical exercise performed by female PWDs in South Korea vary according to exercise type, duration, frequency and degree and participant disability type and age, and discusses ways to enhance the effects of physical exercise. The objective is to not only seek practical ways to increase the efficacy of the physical exercises performed by South Korean female PWDs, but also to make practical suggestions to countries at a similar level to that of South Korea in terms of disabled women’s welfare to help them develop effective exercise programs. In addition, given that female PWDs are less inclined to express their opinions about their desires for exercise in the social structure, the meta-analysis findings are thought to be capable of contributing to the preparation of a scientific basis for female PWDs to perform physical exercise necessary within the community by scientifically presenting the resultant effects of multiple exercises. To this end, the following research questions were set.

1. What is the overall effectiveness of physical exercises performed by female PWDs as a dependent variable?
2. What are the effect sizes by type of physical exercise (weight-bearing exercise, resistance exercise and aerobic exercise) performed by female PWDs?
3. What are the effect sizes for physical exercise among female PWDs by individual characteristics (disability type and age)?
4. What are the effect sizes for physical exercise among female PWDs by program characteristics (duration, period and frequency)?

## 2. Methods

According to the Systematic Reviews and Meta-Analyses (PRISMA) guidelines [30], the procedure for data collection in a systemic literature review involves study identification, study selection, review of inclusion criteria and finalization.

### 2.1. Inclusion Criteria

South Korean studies that assessed the effects of exercise among female PWDs in South Korea were collected and analyzed. The study employed a Population, Intervention, Comparison, Outcomes and Study (PICOS) design using a structured questionnaire to clarify key concepts of the research topic [31]. The study participants (P) were female PWDs, the intervention program (I) was exercise for female PWDs, comparisons (C) were made between female PWDs who received treatment and those who did not, the outcome (O) of intervention was the effectiveness of exercise and the study design (S) was a control group pre-test–post-test design.

### 2.2. Exclusion Criteria

In this study, to mitigate the problem with the representativeness of study findings when low-quality studies are combined in the meta-analysis, only those papers listed in the Korea Citation Index (KCI) were included in the meta-analysis. The criteria for the exclusion of papers were as follows: papers that did not include exercise treatment for female PWDs, papers not on the subject of female PWDs, papers that presented insufficient data showing the effects of exercise treatment for female PWDs and qualitative studies.

### 2.3. Search Strategy

Papers published from 2000 to 2020 were searched. This study searched for studies published on female PWDs in academic journals using the search engine of the Korea Education Research Information Service (RISS) and National Assembly Library of Korea (NALK). Search keywords were ‘women with disabilities’ and ‘women with disabilities, exercise’.

### 2.4. Study Screening and Selection

**Figure 1:**
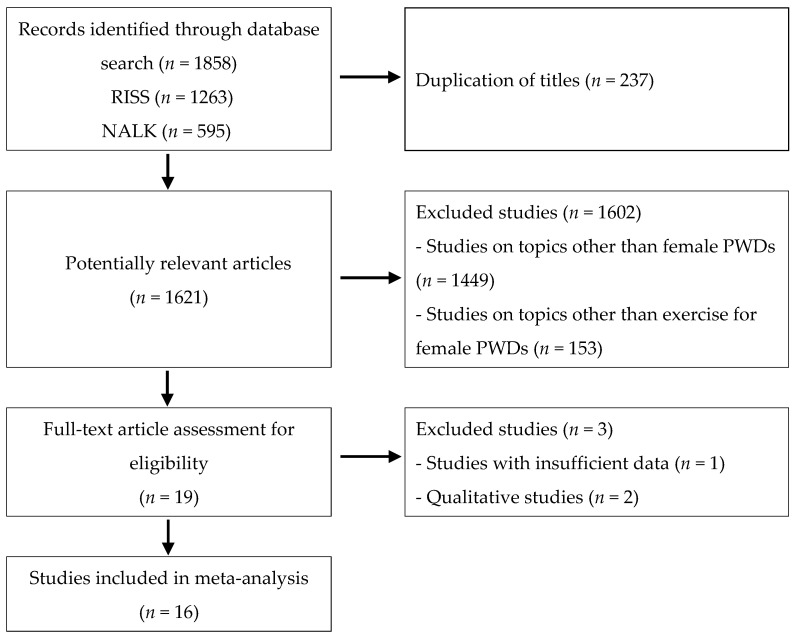
PRISMA flow diagram.

In this study, two reviewers independently performed the procedures shown in Figure 1, and in cases where there were any objections in relation to the study review and selection, they were resolved by agreement through consultation. Figure 1 contains the PRISMA flow diagram of the search process.

The search yielded 1263 and 595 studies from RISS and NALK, respectively, making 1858 journal articles in total. The studies were reviewed using the procedure described in Figure 1. Among the studies obtained from the database search, 237 papers with overlapping titles were excluded. Of the 1621 potentially relevant articles, 1602 studies that were not on the topic of female PWDs or exercise for female PWDs were excluded based on a review of titles and abstracts.

After undergoing the above-mentioned procedure, from among the 19 papers, 3 papers with insufficient data or qualitative methods were excluded. Finally, 16 studies were selected for the meta-analysis.

### 2.5. Data Extraction 

Studies were coded for analyses of effect. Coding items included basic information about the study (title, publication year and sample size), research design (independent and dependent variables and measures) and associated variables. Coding was conducted by a researcher with meta-analysis experience and reviewed by a co-investigator according to agreed-upon guidelines. When individual studies required specific review, they were re-examined, and coding changes were made. After repeating this process, researchers reached an agreement on coded outcomes.

### 2.6. Effect Size

Effect size is a unit used for the synthesis and comparison of study results. Commonly used effect sizes include the standardized mean, correlation coefficient (*r*) and odds ratio.

In the present study, effect sizes were compared using the standardized mean change difference between a group of female PWDs that received exercise treatment and a group that did not. The standardized mean difference was the difference between before and after treatment in the treatment and control groups [32]. Study results are interpreted based on their effect sizes. An effect size smaller than 0.2 indicates a ‘small effect’, one of approximately 0.5 indicates a ‘medium effect’ and one 0.8 or larger indicates a ‘large effect’ [33]. Analyses were performed using CMA 3.0 software.

### 2.7. Publication Bias and Qualitative Evaluation

In this study, publication bias and qualitative evaluation were investigated in order to prevent biased results. The trim and fill method was used to analyze publication bias. As for the quality of individual studies, a qualitative evaluation was conducted, referring to [34], to examine the study validity, indicating how well certain study results measured actual effects, and internal validity, related to minimizing the bias. Methodological validity was evaluated by two independent reviewers for cross-evaluation of the target papers. The quality of each study was assessed as applicable, not applicable or unclear according to nine criteria. One point was obtained for each criterion if the following questions could be answered in the affirmative for the study: (1) Was the sample frame appropriate to address the target population? (2) Were study participants recruited in an appropriate way? (3) Was the sample size adequate? (4) Were the study subjects and setting described in detail? (5) Was data analysis conducted with sufficient coverage of the identified sample? (6) Were valid methods used for the identification of the condition? (7) Was the condition measured in a standard, reliable way for all participants? (8) Was there an appropriate statistical analysis? (9) Was the response rate adequate and, if not, was the low response rate managed appropriately?

## 3. Results

### 3.1. Publication Bias and Qualitative Evaluation

Studies that present statistically significant positive results are more likely to be published than those that do not [35]. Generally, in the absence of publication bias, studies are distributed symmetrically around the aggregated effect size; in the presence of publication bias, studies are clustered to either end.

**Figure 2:**
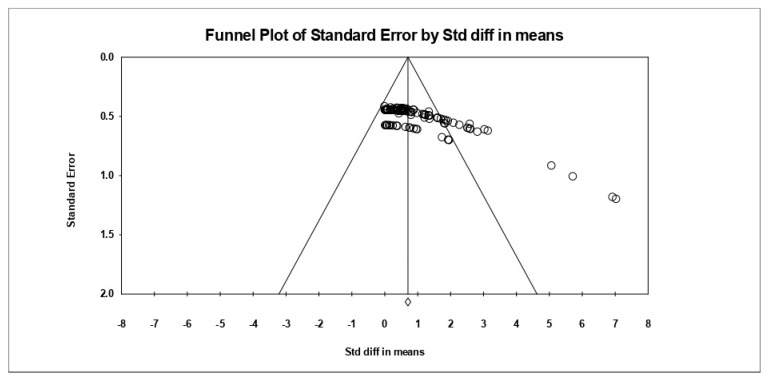
Funnel plot of standard error by standard difference in means.

In Figure 2, the x- and y-axes represent the effect size and standard error, respectively. The funnel plot created to determine publication bias shows a largely symmetrical shape, suggesting that publication bias is not strong. To analyze publication bias errors, the influences of the missing studies were estimated using the trim and fill method. When 10 studies were added to the left in the trim and fill method, the adjusted mean effect size was 0.78, which was about 11.4% higher than the observed effect size of 0.70. The 95% confidence interval of the adjusted mean effect size was 0.72–0.74, which was still statistically significant. Therefore, when these results are taken together, it can be seen that there is a tendency of publication bias, but the bias is not serious enough to overturn the research results. The trim and fill method is a sort of sensitivity analysis, and according to the results, the influences were not large enough to hinder the validity of the estimation of the effects of exercise, as shown in Figure 2.

According to the results of the qualitative evaluation, in two out of 15 papers, the sample frame was not adequate to handle the target recruitment. Regarding participant recruitment, in 10 papers, they were not recruited in an appropriate way, and in 3 papers, it was unclear. In seven papers, the sample size was inappropriate, and in two papers, the explanation of the research topic was inadequate (Supplementary Table S2).

### 3.2. Heterogeneity Test

In a meta-analysis, fixed-effects and random-effects models are used to calculate the mean effect size [36]. A fixed-effects model estimates the effect sizes of the same population, and a random-effects model estimates the mean of the distributions of effect sizes of different populations.

The null hypothesis in the homogeneity test was used to determine the presence of homogeneity. In Table 1, the Q-value represents the observed variance of effect sizes. The degree of freedom (df) is the expected variance when the effect sizes of the populations from individual studies are identical. If the value obtained by subtracting df from the Q-value was a positive value (i.e., Q > df), the effect sizes of populations in individual studies were considered different.

In the present study, the Q-value was 342.423 (Table 1), which is larger than the df of 126, suggesting that the population effect sizes are different. The probability of significance (*p*-value) of the homogeneity test was <0.10; therefore, the heterogeneity of the effect sizes was significant. This suggests that the random-effects model was appropriate. I-squared represents a ratio of actual variance to total variance: 25%, 50% and 75% indicate a small, moderate and very large level of heterogeneity, respectively. In this study, I-squared was 63.311, suggesting heterogeneity among the populations.

Prior to measuring the overall effect size, this study tested homogeneity to determine whether the results of individual studies were extracted from the same population. That is, it evaluated whether the effect sizes derived from individual studies could be regarded as being derived from the same population. In the homogeneity test, if individual studies are extracted from the same population and construed to be homogeneous while the results are statistically significant, effect sizes are estimated based on the random-effects model, which assumes the results of individual studies are from different populations. Under the random-effects model, studies with a small sample size are given a larger weight, while studies with a large sample size are given a smaller weight. The method of Hedge and Olkin [37] was used to assign weights according to the study sample size.

### 3.3. Study Characteristics

In this study, a total of 16 papers were included in the meta-analysis, and the number of participants was 154. The first studies on the effects of exercise performed by female PWDs were [38,39,40]. According to the papers included in the meta-analysis, diverse exercises were performed by female PWDs, including the following: weightlifting and squats as resistance exercises [38]; sitting volleyball [41,42,43], wheelchair tennis [44,45], horseback riding [46,47], badminton [48], yoga [49] and Korean dance [50] as weight-bearing exercises; and aerobics [51], swimming [52,53] and aquatic exercise [38] as aerobic exercises. The effects of such exercises were found to be changes in muscle strength, body composition, physical fitness, psychology, inflammatory factors and blood lipids. The female PWDs who participated in exercises had stroke hemiplegia, mental disorders, physical disabilities, intellectual disabilities and visual impairments, and the age distribution was shown to be diverse, ranging from female PWDs in their 20s to 60s. The averages were as follows: number of persons who participated in an exercise: 9.6, exercise duration: 49 min, frequency of exercise per week: 3.25, exercise period: 12.3 weeks and total number of sessions: 38.8.

Table 2 shows the effect sizes of studies included in the meta-analysis according to the above criteria, participants’ disability type and age, exercise effect and type and program characteristics.

### 3.4. Overall Effect Size

The overall effect size in this study was verified through the random-effects model because individual studies were found to be heterogeneous. The overall effect size was 0.808, and the 95% confidence interval was 0.665–0.950 (Table 3). According to Cohen’s [34] guidelines for the interpretation of effect size based on the difference in standardized correlation (r), r ≤ 0.10, r = 0.40 and r ≥ 0.40 indicate small, medium and large effect sizes. In the analysis, the overall effect size of the correlations for exercise for female PWDs was large.

### 3.5. Effect Size of Exercise for Female PWDs by Exercise Outcome and Type 

Table 4 shows the effect sizes for the outcomes of exercise among female PWDs. The effect was largest in changes in muscle strength (d = 2.407), followed by psychological changes (d = 1.325). Effect sizes for changes in body composition (d = 0.336) and blood lipids (d = 0.370) were small. The effect of exercise was largest in horseback riding (d = 3.080), followed by swimming (d = 1.648).

### 3.6. Control Group

In addition to the effect of exercise treatment in the papers included in the meta-analysis, regarding the control group, Song [38] examined the effects of exercise treatment with a control group that performed only underwater resistance exercise and an experimental group that performed underwater exercise.

In Ahn et al. [39], the subjects were randomly assigned to a control group that received only physical therapy and an exercise group for which physical therapy and elastic band exercises were combined. In Choi and Jang [50], those who were able to perform exercise were assigned to the experimental group, and those who found it difficult to perform exercise were assigned to the comparison group. In the remaining 13 papers included in the meta-analysis, the subjects were randomly assigned to the experimental and control groups, but the treatment(s) applied to the control group was not clearly indicated.

### 3.7. Effect Size by Individual Characteristics of Female PWDs

Table 5 shows the effect size by individual characteristics of female PWDs. Regarding disability type, the effect size was largest among women with visual impairments (d = 3.080). Regarding age, the effect size was largest among women in their 40s (d = 1.390).

### 3.8. Effect Size by Program Characteristics of Exercise among Female PWDs

Table 6 shows the effect size by program characteristics of exercise among female PWDs. Regarding duration of sessions, the effect size was largest for durations of 45–50 min (d = 3.080). Regarding frequency, the effect size was largest for a frequency of three times a week (d = 0.963). Regarding the period, the effect size was largest for 15-week programs (d = 1.974). Regarding the total number of sessions, the effect size was largest for 45 sessions (d = 1.974).

## 4. Discussion

The present study investigated the effects of physical exercise and their implications among female PWDs using a meta-analysis. Specifically, the effect sizes of physical exercises for female PWDs were compared by gathering the results of existing studies to examine the effect sizes of correlations. The meta-analysis included results from 16 studies.

First, the results were examined in relation to the effect of physical exercise treatment of female PWDs and the effect sizes by exercise type. The effect size for the outcome of physical exercise was largest in changes in muscle strength, followed by psychological change. Both psychological and physical changes must be examined, and these changes are demonstrated through the results of this meta-analysis. The findings are in line with the idea that the lives of PWDs are multifaceted and affected by a multitude of circumstances, including societal, historical, family and community values. Additionally, exercise programs should include PWDs in this broad context [54].

Regarding the type of physical exercise, the effect size was largest for horseback riding, followed by swimming classes and swimming programs. Horseback riding is challenging in terms of accessibility and affordability, while swimming is highly accessible. Furthermore, swimming is in high demand because it improves quality of life and leisure time while promoting health and enhancing physical strength. Kim and Kim [28] found a high rate of participation in swimming among female PWDs, which suggests the need for strategies to promote swimming among these populations. By contrast, the effect sizes were small for wheelchair dance, water exercise and aerobic exercise. Durstine et al. [55] suggested that when prescribing exercise, clinical condition should be emphasized. Therefore, the mode, intensity, frequency and duration of exercise should be modified according to the individual’s clinical condition. These results suggest the importance of physical exercise that incorporates individual characteristics by demonstrating that female PWDs need to select a physical exercise that is suitable for their environment and characteristics [56].

Second, regarding the effects by personal characteristics of female PWDs, effect sizes by disability type and age were examined. Regarding the type of disability, the effect size was largest among women with visual impairments. This may be because women with visual impairments have fewer physical difficulties than do women with other disabilities. The effect sizes were relatively small for mental disorders and intellectual disabilities. Top et al. [57] studied the effects of swimming in people with mental disorders and found no statistically significant differences between the treatment and control groups. This is consistent with the findings of this study. This may be related to difficulties associated with exercising among women with intellectual disabilities. Thus, exercise equipment or programs that can be easily used by women with intellectual disabilities are required to improve physical exercise efficacy among this population.

Regarding age, the effect size was largest among female PWDs in their 40s, followed by those in their 30s, 20s and 50s. This does not show a clear correlation between age and the effects of physical exercise. Additionally, the meta-analysis suggested that research on female PWDs in their 60s is lacking, despite its growing importance with the phenomenon of increased life expectancy in South Korean society.

Third, regarding the effects by physical exercise composition for female PWDs, effect sizes by the duration of one physical exercise session, frequency of exercise per week, physical exercise period and total number of physical exercise sessions were examined. Regarding the duration of physical exercise sessions, the effect of physical exercise for female PWDs was largest when a session lasted 45–50 min. By contrast, the effect size was relatively small for 60 min sessions. The results suggest that the effect of physical exercise is greater when the duration of a session is up to 60 min. Regarding the frequency of sessions, the effect size was largest for sessions that occurred three times a week. The effect of exercise was smallest when sessions occurred once or five times a week.

Regarding the period, the effect size was largest when exercises ran for 15 weeks. Son et al. [58] found that physical exercise for PWDs has the greatest effects when it lasts for at least 10 weeks. The findings of that study demonstrate the importance of an extended period of physical exercise. Interestingly, in the present study, the effect size was smaller when the period of exercise was over 15 weeks. Regarding the total number of physical exercise sessions, the effect size was largest when the physical exercise lasted for 45 sessions in total. These results suggest that the effect is largest when exercise lasts for approximately 3 months rather than for a short-term period. Furthermore, exercising for either much less or much more than 15 weeks is ineffective.

The significance of the findings of this study can be divided into three points. First, this study can be considered to have theoretically contributed to the literature on exercise performed by female PWDs in that the findings of this study indicate that exercise performed by female PWDs has not only psychological effects, but also physical effects, and that the exercise effects of swimming using community spaces are large. This supports the notion that exercise performed by women with disabilities should be seen not only from the perspective of recovery from physiological damage, but also from a social perspective.

Second, the findings of this study suggest that other exercise programs should be supported in order to enhance the physical exercise effects of the physically, mentally or intellectually disabled. This can be said to have laid a foundation for separately planning exercise programs for the mentally disabled and for the intellectually disabled.

Third, this study can provide practical help in composing exercise programs for female PWDs because it concretely presents the duration, number of sessions and period appropriate for physical exercise effects in South Korea or in countries with similar welfare dimensions or cultural characteristics for female PWDs.

In summary, these study results suggest that individual characteristics and environment affect the optimal level of exercise.

## 5. Conclusions

In terms of limitations, since this study was conducted with a relatively small volume of study results, the generalizability of the study findings is limited. Therefore, it is thought that this study should be supplemented by follow-up studies when more studies on exercise programs for female PWDs are accumulated. In addition, there were limitations in comparing the effects of exercise according to gender, as studies on PWDs were conducted without distinguishing between men and women. This study investigated the effects of exercise among South Korean female PWDs and included no comparison with studies on male PWDs. Therefore, further research must be conducted among male PWDs to investigate the effect size of exercise for these PWDs.

The study also lacked an analysis of the characteristics of South Korean culture in relation to exercise among female PWDs. In the future, studies should be conducted on exercise participation and opportunities for female PWDs in the context of South Korean culture. In addition, studies have shown that the reason for participating in sports or physical activity for long periods of time is the enjoyment of the activity itself rather than for maintaining health or physical training [25,59,60,61]. In the present study, it was challenging to examine the enjoyment of exercise. To maximize the effects of exercise among female PWDs, various factors need to be examined. Finally, while this study made a unique contribution by analyzing the effect sizes of studies with treatment and control groups via a meta-analysis, further research is needed to investigate the subjective experiences of female PWDs by including qualitative studies on exercise for the population in a meta-analysis.

In terms of conclusions, first, there is a lack of studies on female PWDs in South Korea. Regarding the theory of PWDs, studies on exercise performed by PWDs should apply either the medical model or the social model or both in combination. In South Korea, disabilities cause more difficulties to women when they lead their social lives. Therefore, it is more necessary to enable female PWDs to perform their activities more smoothly through physical exercise and to create conditions for their social activities. In addition, conditions under which female PWDs in South Korea can exercise more easily should be prepared. While the South Korean economy has grown rapidly, the budget for PWDs’ welfare is relatively insufficient. The problems of female PWDs should be seen as not only personal problems, but also important social and national issues, and the support process and system for female PWDs should be strengthened further.

Second, it is worth noting that the correlation between the effect sizes and exercise treatments for female PWDs was strong and that the effect sizes by type of exercise treatment were large not only in terms of psychological changes, but also in terms of muscle strength. Therefore, it is necessary to develop and implement comprehensive exercise programs that can bring about physical and psychological changes through exercise performed by female PWDs in the future.

Third, regarding the sizes of exercise effects by personal characteristics of female PWDs, the effects of exercise on the visually impaired were shown to be the largest, but the effects on women with mental disorders or intellectual disabilities were shown to be small. These findings can be said to be related to the physical/mental conditions for performing exercise. Therefore, it is necessary to develop exercise programs suitable for individual disability characteristics. Meanwhile, regarding exercise effect sizes by age, although exercises performed by female PWDs in their 40s showed large effects, effects were relatively small for those in their 20s and relatively large for those in their 30s and 50s. Therefore, exercise effects could not be considered to be correlated with age. These findings show that further studies on the correlation between age and exercise effects are necessary and that exercise programs must be developed and implemented by age.

Finally, the effect sizes were largest when a session lasted 45–50 min, the frequency of sessions was three sessions per week, the period of exercise was 15 weeks and the total number of sessions was 45. In summary, the effect of exercise was greatest when PWDs exercised a total of 45 times over 15 weeks at three sessions per week and 45–50 min per session. This suggested that the effect of exercise among female PWDs is largest when they exercise every other day for approximately 3 months.

## Figures and Tables

**Table 1:** Test of heterogeneity.

Model	Q-Value	*df* (Q)	*p*-Value	I-Squared
Fixed	343.423	126	0.000	63.311

**Table 2:** Characteristics of included studies.

No.	Researcher	Effect Size	Participant’s Disability Type	Participant’s Age	Number of Participants	Exercise Type	Effect of Exercise	Duration of Exercise (min)	Frequency of Exercise/per Week	Period of Exercise (Weeks)	Total Number of Sessions
1	Song (2009)	0.866	Physical disability	Not indicated	6	Water exercise	Changes in body composition	60	5	8	40
2	Ahn et al. (2009)	0.975	Hemiplegia after stroke	50s and 60s	6	Elastic band exercise	Changes in physical strength	50	6	6	36
3	Kim & Lim (2009)	1.037	Mental disorder	40s and 50s	10	Aerobic and gym ball exercise	Changes in body composition	50	3	16	48
4	Park et al. (2010)	1.011	Physical disability	40s and 50s	11	Sitting volleyball	Changes in muscle strength	120	3	12	36
5	Choi & Jang (2010)	0.838	Intellectual disability	30s and 40s	10	Korean dance	Changes in body composition	60	1	8	8
6	Gwon (2011)	0.598	Intellectual disability	20s	11	Aerobics	Changes in body composition	40	4	12	48
7	Park (2011)	0.558	Mental disorder	40s and 50s	10	Yoga	Changes in physical strength	50–60	3	12	36
8	Lee (2011)	0.669	Physical dysfunction and joint disorder	40s	10	Wheelchair tennis	Changes in muscle strength	60	3	12	36
9	Kim & Park (2012)	0.627	Physical disability	40s	10	Sitting volleyball	Changes in body composition	60	3	12	36
10	Lee (2012)	0.821	Physical disability	40s	10	Wheelchair dance	Changes in blood lipids	60	3	24	72
11	Kim et al. (2013)	0.537	Visual impairment	40s	10	Horseback riding	Changes in muscle strength	45–50	3	12	36
12	Lee & Kim (2015)	0.903	Physical disability	40s	11	Swimming	Psychological change	50	3	15	45
13	Lee & Lee (2015)	0.589	Visual impairment	40s	10	Horseback riding	Psychological change	45–50	3	12	36
14	Kim (2018)	1.319	Physical disability	30s and 40s	10	Sitting volleyball	Psychological change	60	3	16	48
15	Kim (2018)	1.748	Physical disability	40s	10	Swimming	Psychological change	60	3	12	36
16	Kang & Kim (2020)	0.088	Physical disability	30s and 40s	9	Badminton	Psychological change	60	3	8	24

**Table 3:** Overall effect size and random-effects analysis.

	*k*	ES	95% CI		Q-Value	df	*p*-Value	I^2^
			Lower	Upper				
Total	127	0.808	0.665	0.950	343.423	126	0.000	63.311

Note: *κ* = number of samples; ES = effect size; Q = observed variance of effect sizes; df = degree of freedom; I^2^ = heterogeneity (ratio of actual variance to total variance).

**Table 4:** Effect size of exercise outcome and type.

Category	*k*	*d*	SE	95% CI	
				LL	UL
Exercise outcome					
Changes in muscle strength	12	2.407	0.473	1.481	3.334
Changes in body composition	28	0.336	0.088	0.164	0.508
Changes in physical strength	25	0.545	0.108	0.332	0.758
Psychological change	37	1.325	0.183	1.013	1.637
Mental health change	7	0.491	0.164	0.170	0.812
Inflammatory factor changes	6	0.422	0.183	0.064	0.780
Changes in blood lipids	12	0.370	0.131	0.113	0.626
Exercise type					
Strength exercise	6	0.423	0.185	0.060	0.785
Badminton	6	1.194	0.228	0.747	1.641
Swimming	10	1.648	0.304	1.051	2.245
Water exercise	4	0.292	0.292	0.279	0.864
Horseback riding	13	3.080	0.408	2.280	3.881
Yoga	9	0.358	0.151	0.063	0.653
Aerobic exercise	20	0.304	0.121	0.068	0.541
Sitting volleyball	24	0.625	0.108	0.413	0.836
Elastic band exercise	16	0.742	0.170	0.409	1.074
Korean dance	6	0.090	0.183	0.268	0.448
Wheelchair dance	8	0.276	0.160	0.037	0.588
Wheelchair tennis	5	1.238	0.220	0.806	1.670

Notes: *k*, number of samples; *d*, Cohen’s d effect size; SE, standard error; 95% CI, 95% confidence interval, LL, lower limit, UL, upper limit.

**Table 5:** Effect size by individual characteristics.

Category	*k*	*d*	SE	95% CI	
				LL	UL
Disability type					
Hemiplegia after stroke	16	0.742	0.170	0.409	1.074
Visual impairment	13	3.080	0.408	2.280	3.881
Mental disorder	23	0.325	0.094	0.141	0.510
Intellectual disability	18	0.307	0.105	0.100	0.513
Physical dysfunction	5	1.238	0.220	0.806	1.670
Physical disability	52	0.798	0.099	0.605	0.991
Age					
20s	12	0.415	0.129	0.162	0.668
30s–40s	15	0.886	0.216	0.464	1.308
40s	46	1.390	0.169	1.058	1.722
40s–50s	34	0.404	0.077	0.254	0.554
50s–60s	16	0.742	0.170	0.409	1.074
Not indicated	4	0.292	0.292	−0.279	0.864

Notes: *k*, number of samples; *d*, Cohen’s d effect size; SE, standard error; 95% CI, 95% confidence interval; LL, lower limit; UL, upper limit.

**Table 6:** Effect size by exercise program characteristics.

Category	*k*	*d*	SE	95% CI	
				LL	UL
Duration of session					
40 min	12	0.415	0.129	0.162	0.668
45–50 min	13	3.080	0.408	2.280	3.881
50 min	35	0.713	0.134	0.450	0.976
50–60 min	9	0.358	0.151	0.063	0.653
60 min	47	0.702	0.095	0.515	0.888
120 min	11	0.558	0.131	0.300	0.815
Frequency per week					
1 session	6	0.090	0.183	−0.268	0.448
3 sessions	89	0.963	0.095	0.776	1.150
4 sessions	12	0.415	0.129	0.162	0.668
5 sessions	4	0.292	0.292	−0.279	0.864
6 sessions	16	0.742	0.170	0.409	1.074
Period (weeks)					
6	16	0.742	0.170	0.409	1.074
8	16	0.533	0.155	0.230	0.837
12	65	0.950	0.109	0.737	1.164
15	5	1.974	0.640	0.719	3.228
16	17	0.555	0.154	0.252	0.857
24	8	0.276	0.160	−0.037	0.588
Total number of sessions					
8	6	0.090	0.183	−0.268	0.448
24	6	1.194	0.228	0.747	1.641
36	69	1.024	0.111	0.806	1.242
40	4	0.292	0.292	−0.275	0.864
45	5	1.974	0.640	0.719	3.228
48	29	0.478	0.091	0.300	0.657
72	8	0.276	0.160	−0.037	0.588

Notes: *k*, number of samples; *d*, Cohen’s d effect size; SE, standard error; 95% CI, 95% confidence interval; LL, lower limit; UL, upper limit.

## Data Availability

Data from the study are available upon request.

## Supplementary Materials

The following are available online at https://www.mdpi.com/article/10.3390/ijerph182312791/s1. Table S1: PRISMA checklist, Table S2: Quality assessment.
